# Supplementary material for: The genomic landscape of TERT promoter wildtype-IDH wildtype glioblastoma
Source: Nat Commun. 2018 May 25;9:2087. doi: 10.1038/s41467-018-04448-6 (PMC5970234; doi:10.1038/s41467-018-04448-6)
Supplement: Supplementary file 1 — Supplementary Information [file 41467_2018_4448_MOESM1_ESM.pdf]

Supplementary Information

The genomic landscape of *TERT* promoter wildtype-*IDH* wildtype glioblastoma

Diplas et al.

a

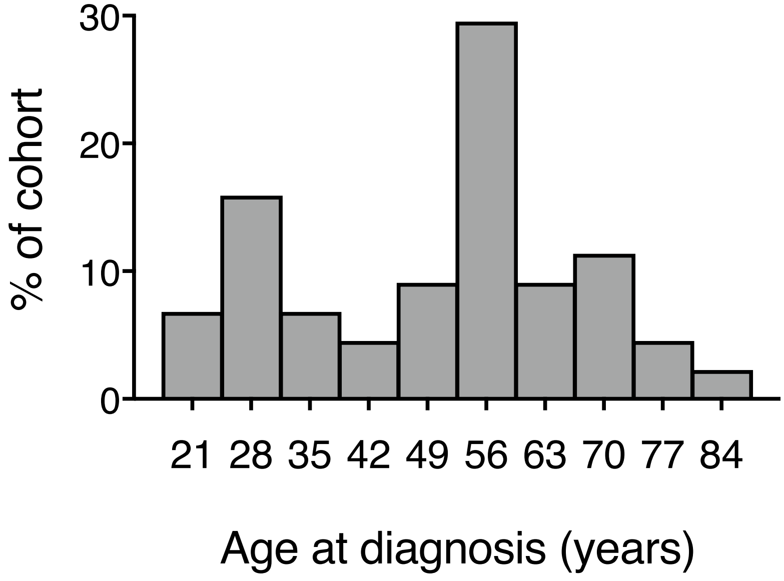

Supplementary Figure 1. Age distribution of *TERTp*^{WT}-*IDH*^{WT} glioblastoma patients. a, The distribution of patient age for the *TERTp*^{WT}-*IDH*^{WT} glioma cohort is shown as a percentage of the entire group. Two modes are identified in the cohort, one at 28 years, the other at 56 years of age (N=44).

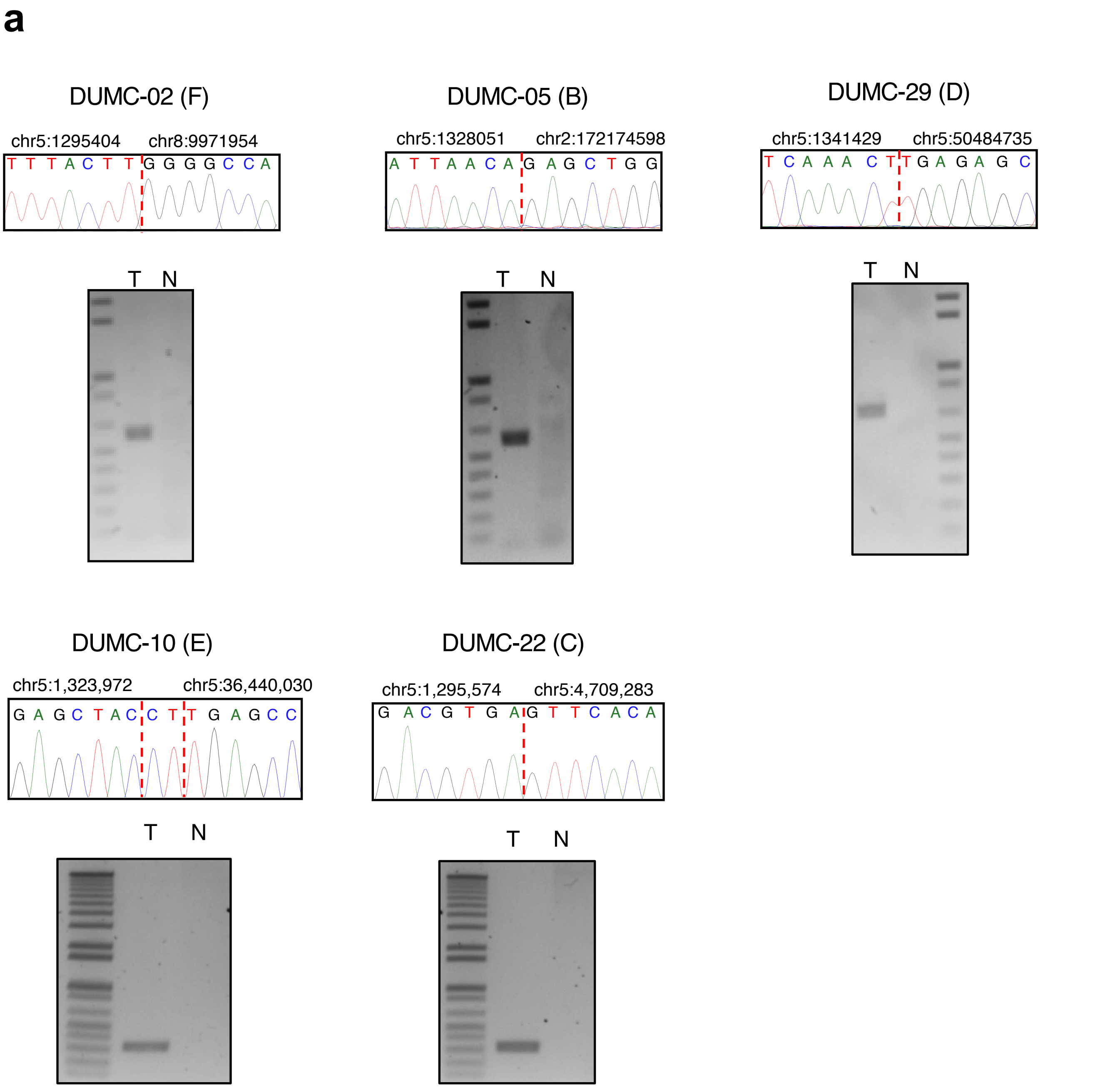

Supplementary Figure 2. Breakpoint-spanning PCR and sequencing confirms *TERT* rearrangements. a, Whole genome sequencing data was analyzed for structural variants. Rearrangements upstream of *TERT* were identified by DELLY, which also identified the corresponding breakpoint. Primers were designed spanning the breakpoints and PCR was performed to confirm the somatic nature of these breakpoints (T: Tumor and N: Normal gDNA from blood for the same patient).

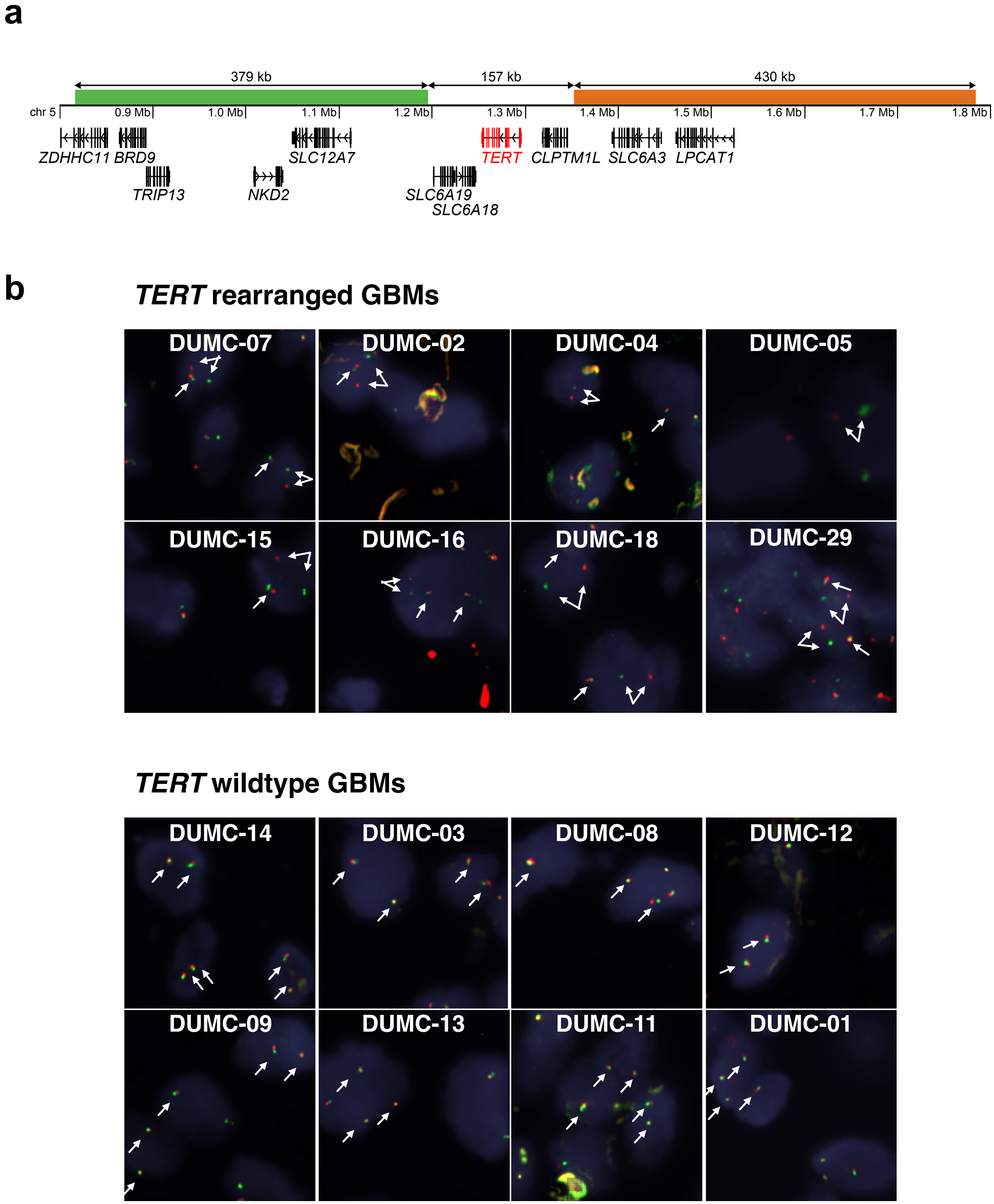

Supplementary Figure 3. Break-apart FISH spanning *TERT* identifies recurrent *TERT* rearrangements in *TERTp*^{WT}-*IDH*^{WT} GBM. a, Break-point spanning FISH probes were designed to readily detect structural variants upstream of *TERT* where we initially identified these events by whole genome sequencing. **b,** Break-apart FISH was performed on FFPE tissue isolated from the GBM patient tumor samples. Representative images from 8 rearranged and wildtype cases are shown. Double arrows point to break apart signals, single arrows point to fusion signals.

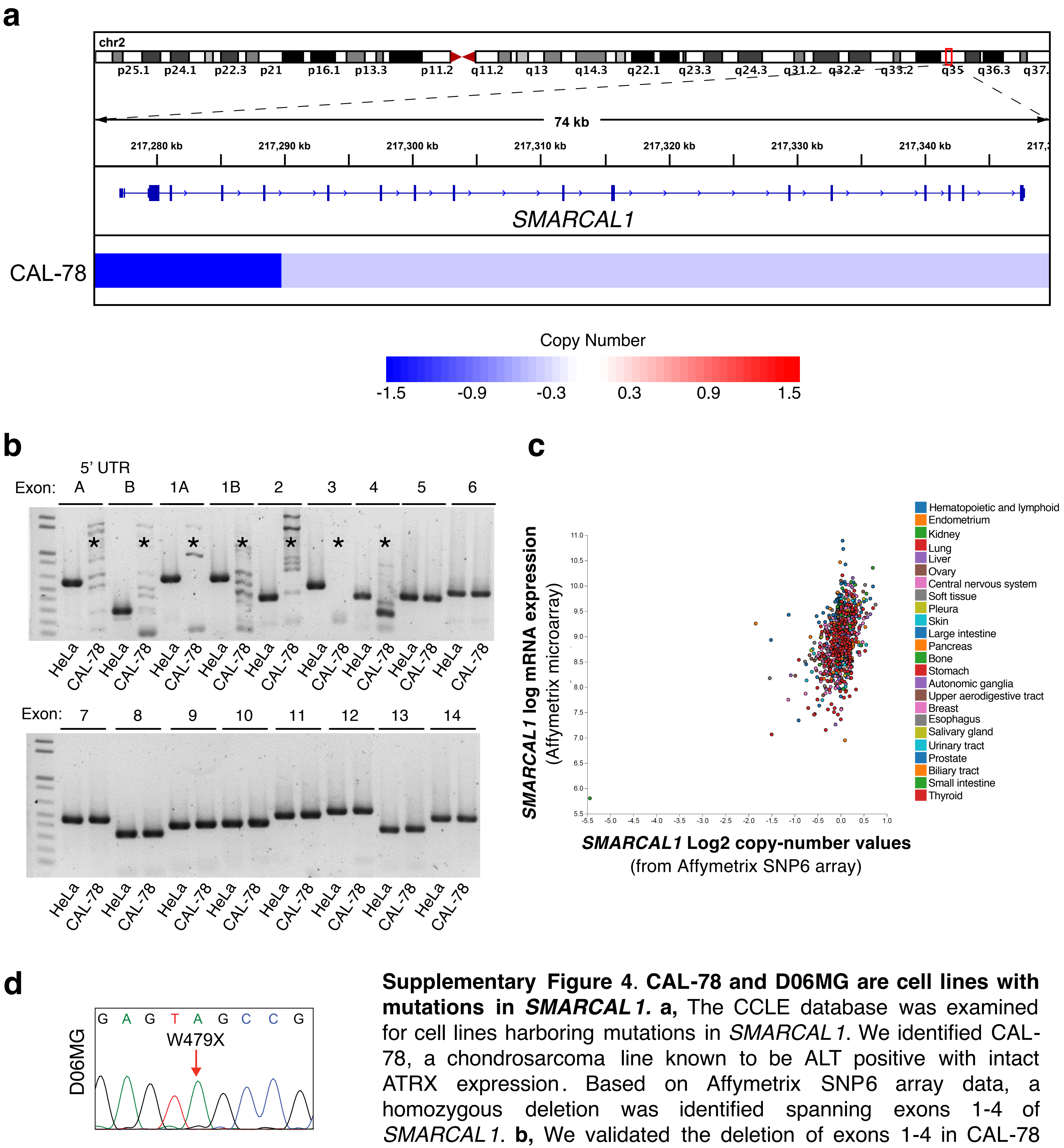

Supplementary Figure 4. CAL-78 and D06MG are cell lines with mutations in *SMARCAL1*. a, The CCLE database was examined for cell lines harboring mutations in *SMARCAL1*. We identified CAL-78, a chondrosarcoma line known to be ALT positive with intact ATRX expression. Based on Affymetrix SNP6 array data, a homozygous deletion was identified spanning exons 1-4 of *SMARCAL1*. **b,** We validated the deletion of exons 1-4 in CAL-78 (including the 5'UTR) by PCR and Sanger sequencing, using HeLa as a control. **c,** Compared to other cell lines, CAL-78 shows both deletion and absent mRNA expression of *SMARCAL1*. **d,** Sanger sequencing of the D06MG cell line reveals a homozygous W479X mutation in *SMARCAL1*.

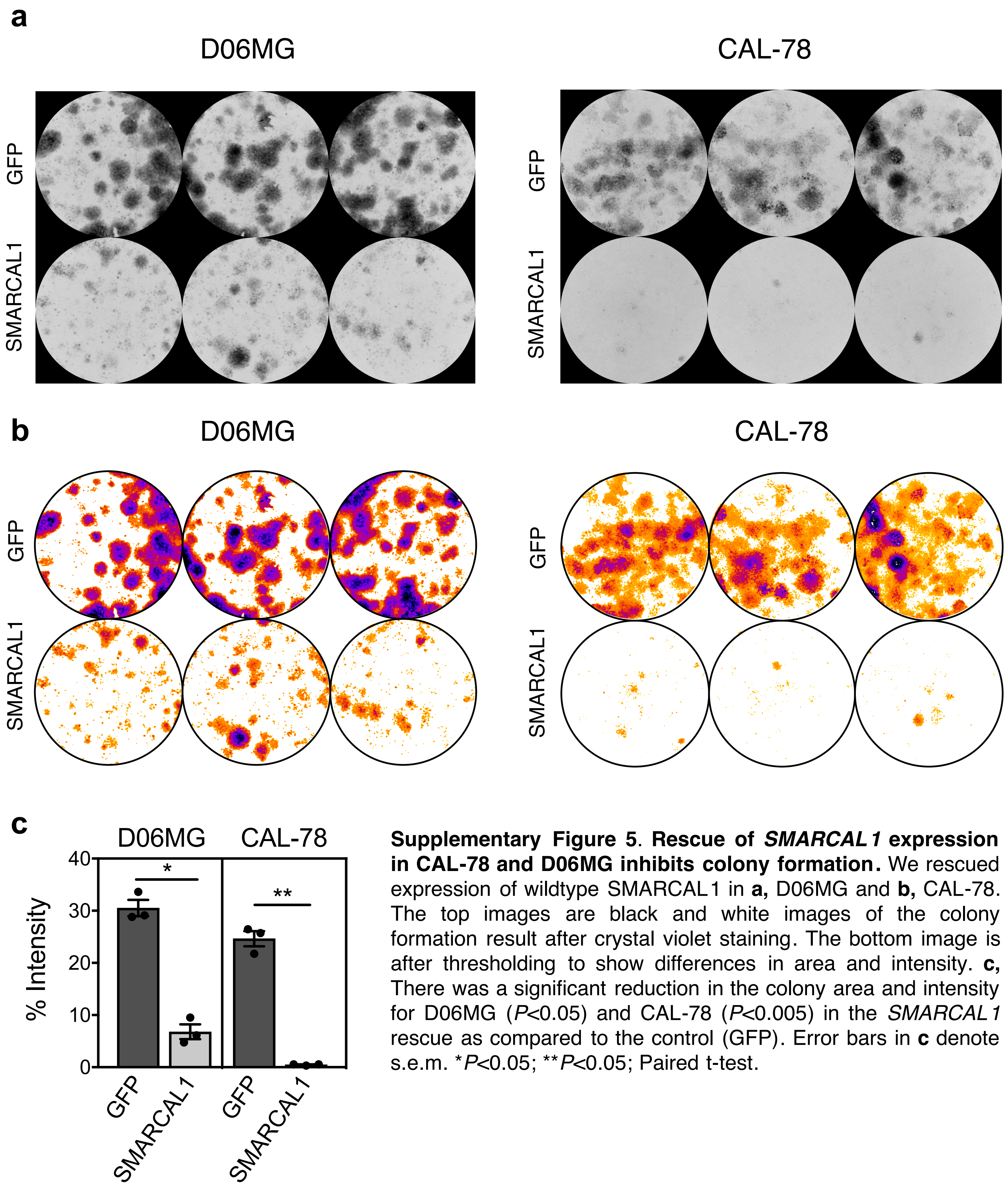

Supplementary Figure 5. Rescue of *SMARCAL1* expression in CAL-78 and D06MG inhibits colony formation. We rescued expression of wildtype SMARCAL1 in **a,** D06MG and **b,** CAL-78. The top images are black and white images of the colony formation result after crystal violet staining. The bottom image is after thresholding to show differences in area and intensity. **c,** There was a significant reduction in the colony area and intensity for D06MG (P<0.05) and CAL-78 (P<0.005) in the *SMARCAL1* rescue as compared to the control (GFP). Error bars in **c** denote s.e.m. *P<0.05; **P<0.05; Paired t-test.

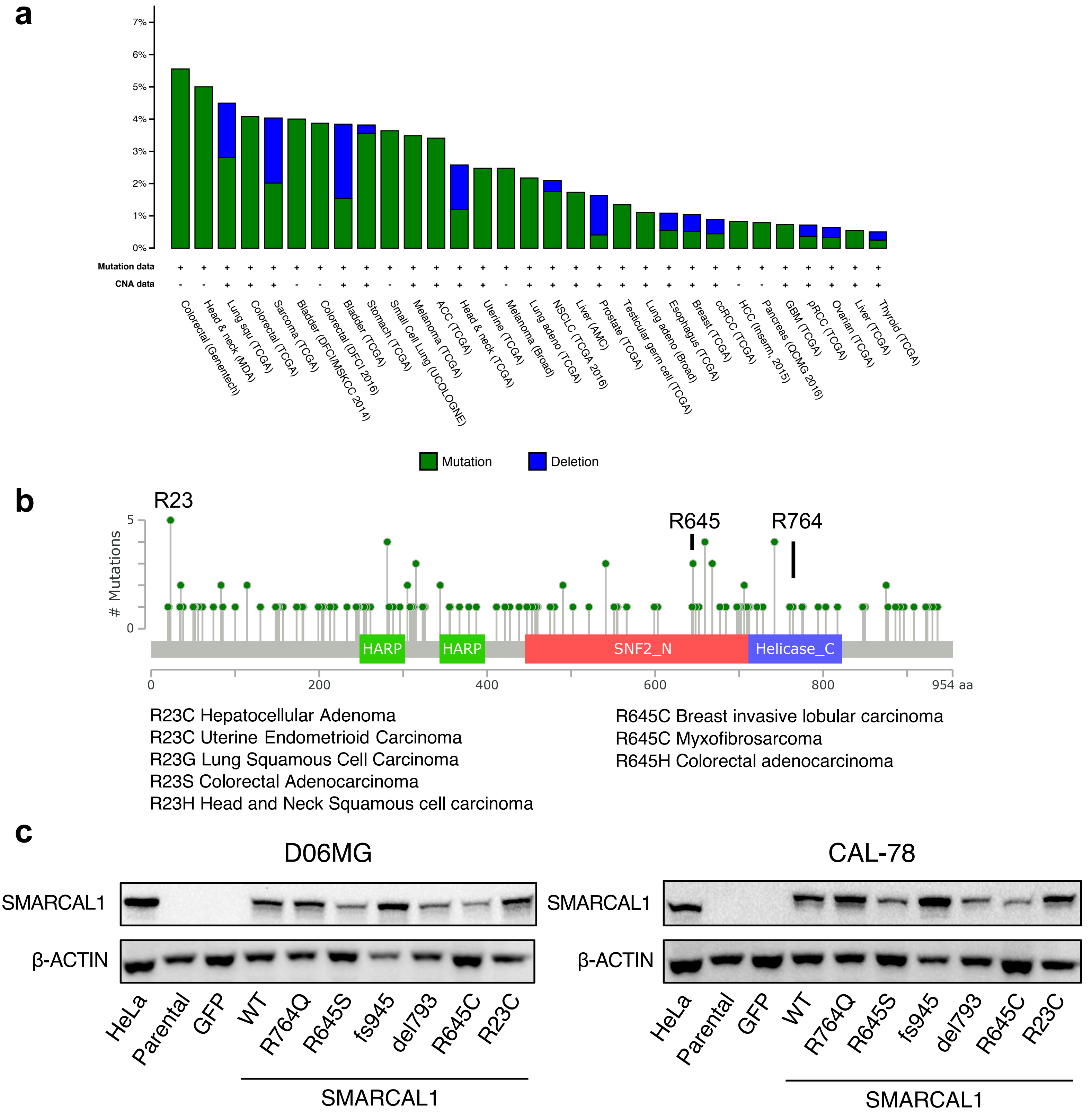

Supplementary Figure 6. Lentiviral-mediated delivery of mutagenized constructs of SMARCAL1 in two ALT-positive lines lacking *SMARCAL1* expression. a, We examined pan-cancer data (cBioportal) for mutations and homozygous deletions. **b,** Recurrently-mutated loci included R23 and a cluster in the SNF2 helicase domain at R645. R23C is located in the RPA-binding domain and forms hydrogen bonds with RPA. R645C is in the ATP-binding helicase domain (also SNF2 N-terminal domain and putative nuclear localization signal domain) and is a known alteration in SIOD. Additionally, from our validation cohort, we identified ALT-positive cases with R645S, del793 and fs945 mutations. **c,** Mutagenized constructs with each of these variants were generated and delivered by lentivirus for constitutive expression in D06MG and CAL-78. Western blot analysis shows that the constructs are expressed at similar levels in these cell lines and similar to a control cell line (HeLa).

a

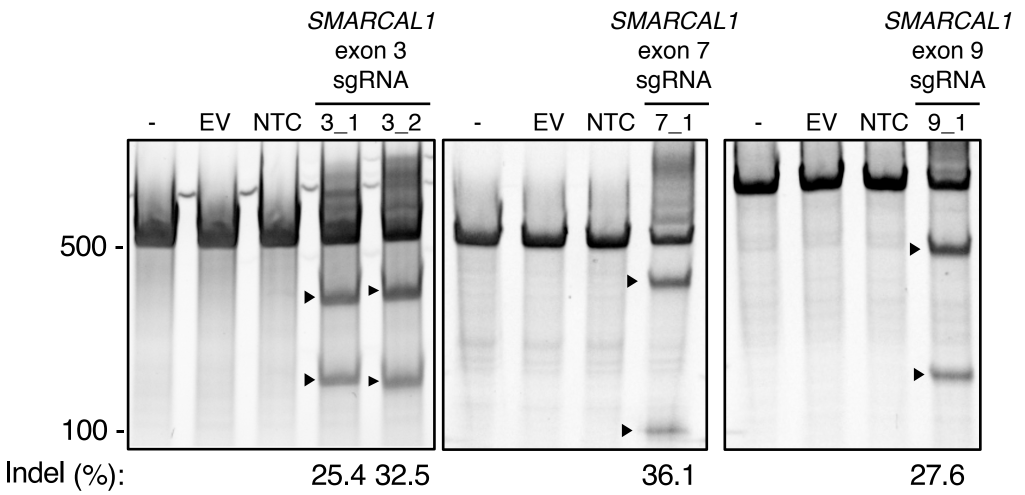

b

				Number of single cell clones:		
**Cell line**	**Guide Set**		**Targeted exons**	**Screened**	**Harboring deletion product**	**Validated knockout**
**U87**	sg*SMARCAL1* A	3_2 + 9_1	3 and 9	83	12	**2**
	sg*SMARCAL1* B	3_1 + 7_1	3 and 7	90	16	**9**
**U251**	sg*SMARCAL1* A	3_2 + 9_1	3 and 9	93	7	**5**
	sg*SMARCAL1* B	3_1 + 7_1	3 and 7	93	22	**7**

Supplementary Figure 7. Generation of *SMARCAL1* knockout glioblastoma cell lines using CRISPR/Cas9-mediated gene editing. **a,** Guides were designed to target the coding region of *SMARCAL1* with low off-targets and high cutting efficiency. The guides were tested using the Surveyor nuclease assay after transfecting HEK293FT cells with pX458 (spCas9) and the relevant sgRNA. All guides readily introduced indels (>20%). **b,** Two guide combinations (A: 3_2 + 9_1 and B: 3_1 + 7_1) were delivered to U87MG and U251MG. After transfection, cells were GFP-sorted and single-cell cloned and expanded. Deletion-spanning qPCR was performed to readily identify clones with allele deletion in *SMARCAL1*. These lines were then sequenced and validated as isogenic knockout lines by Western blot. Overall, more than ten isogenic *SMARCAL1* knockout lines were generated in both U87 (11 total) and U251 (12 total). Clone c69* was excluded from further analysis due to the presence of a faint band by immunoblot.

a

TCGA ID	Cancer Type	Mutation	Mutation Type	CNV Status	Mutant Allele Fraction
TCGA-QC-A6FX-01	Soft tissue sarcoma	T724M	Missense	Shallow Deletion	17.0%
TCGA-DX-AB32-01	Soft tissue sarcoma	R645C	Missense	Shallow Deletion	40.0%
TCGA-JV-A5VF-01	Soft tissue sarcoma	E803G	Missense	Shallow Deletion	59.0%
TCGA-QQ-A5VB-01	Soft tissue sarcoma	K875R	Missense	Shallow Deletion	63.0%
TCGA-IW-A3M6-01	Soft tissue sarcoma	Q145*	Nonsense	Shallow Deletion	66.0%
TCGA-DX-A3U7-01	Soft tissue sarcoma	Homozygous Deletion		Homozygous Deletion	
TCGA-DX-AB2F-01	Soft tissue sarcoma	Homozygous Deletion		Homozygous Deletion	
TCGA-DX-AB2P-01	Soft tissue sarcoma	Homozygous Deletion		Homozygous Deletion	
TCGA-WK-A8XX-01	Soft tissue sarcoma	Homozygous Deletion		Homozygous Deletion	
TCGA-X6-A8C2-01	Soft tissue sarcoma	Homozygous Deletion		Homozygous Deletion	

Supplementary Figure 8. ***SMARCAL1* mutations are present in soft tissue sarcoma**. **a,** We examined a recent TCGA sequencing study on many sarcoma subtypes and found several homozygous deletions and potentially inactivating variants, as many had concurrent shallow deletion and mutations present in the helicase domains.

a

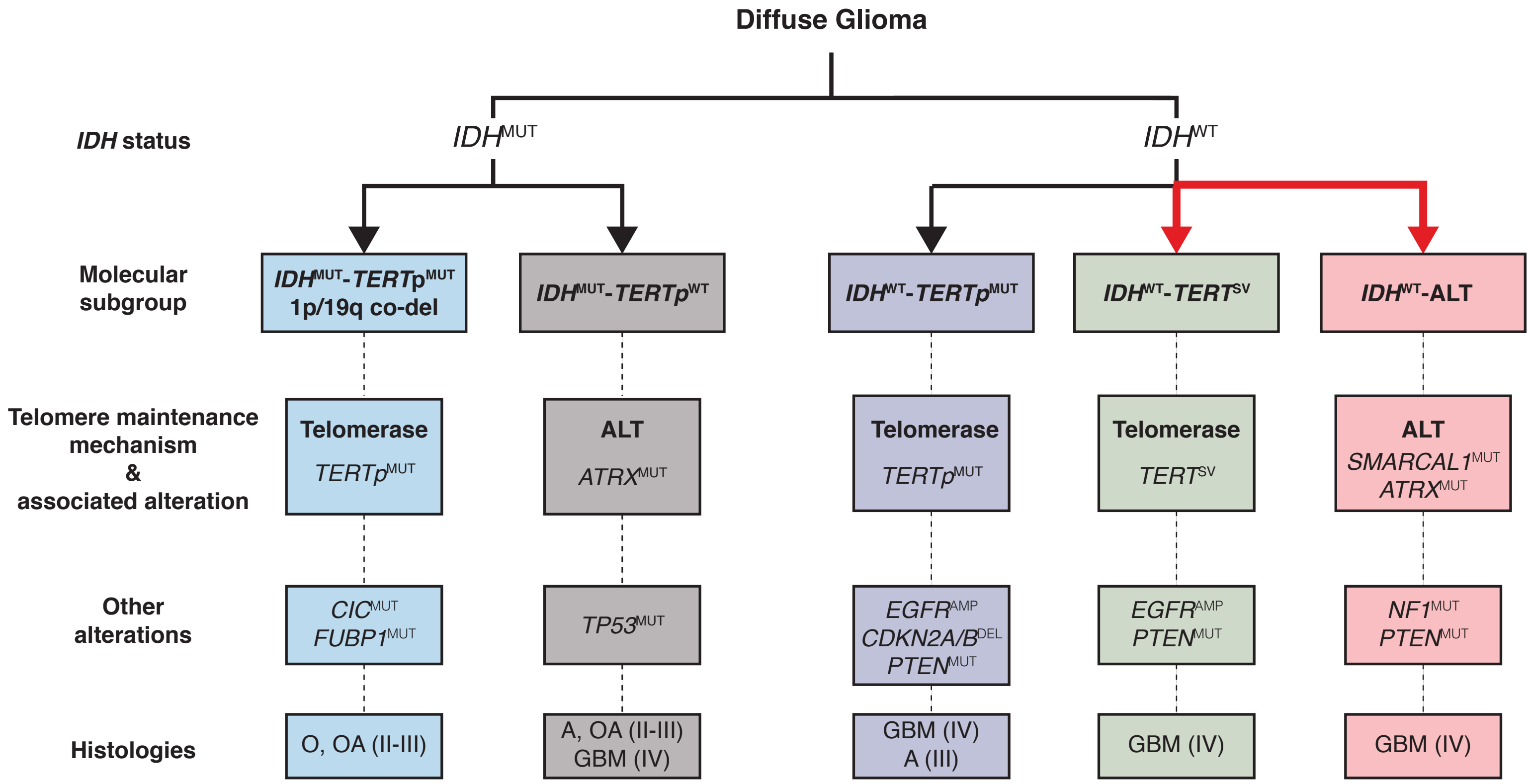

Supplementary Figure 9. Novel genetic subtypes of GBM in the overall molecular classification of adult diffuse glioma. a, The molecular subtypes of GBM are outlined, stratified first by *IDH* status, then by markers including *TERT*p mutation, 1p/19q co-deletion, and now *TERT* rearrangement (*IDH*^{WT}-*TERT*^{SV}) and *SMARCAL1* or *ATRX* mutation (*IDH*^{WT}-ALT). The two new genetic subtypes of GBM, *IDH*^{WT}-*TERT*^{SV} and *IDH*^{WT}-ALT (red arrows), have novel genetic alterations associated with telomere maintenance.

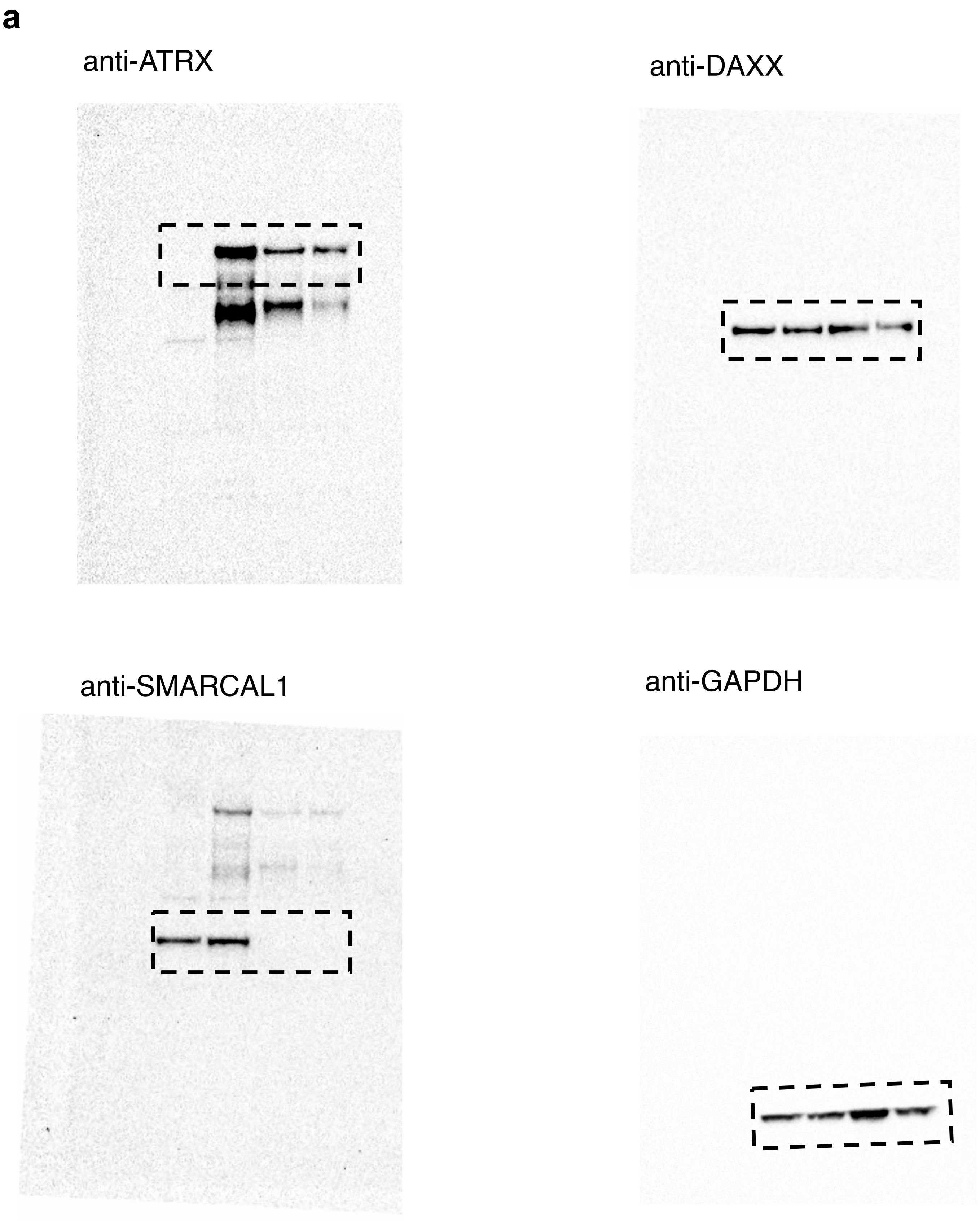

Supplementary Figure 10. Original western blots for examining SMARCAL1 expression in mutant cell lines. a, Shown are the original blots for ATRX, DAXX, SMARCAL1, and GAPDH expression in HeLa, U2-OS, D06MG, and CAL-78. The images are cropped on the right side as unrelated samples were run on the same gel.

a

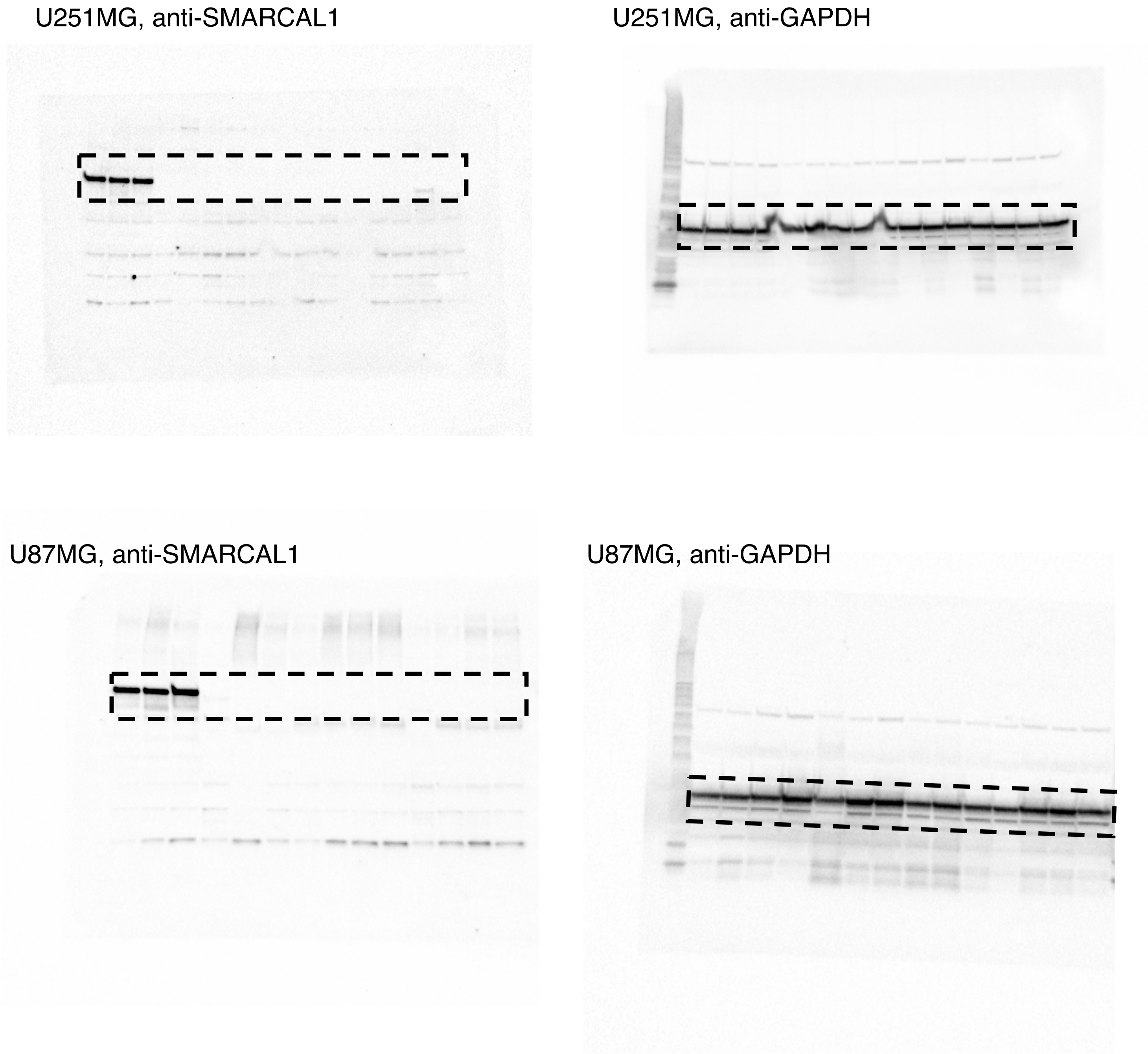

Supplementary Figure 11. Original western blots for SMARCAL1 expression in isogenic knockout cell lines. a, Shown are the original blots for SMARCAL1 and GAPDH expression in U87MG and U251MG *SMARCAL1* knockouts. The images for U87MG is cropped on the right side as unrelated samples were run on the same gel.
