# Supplementary material for: The genomic landscape of TERT promoter wildtype-IDH wildtype glioblastoma
Source: Nat Commun. 2018 May 25;9:2087. doi: 10.1038/s41467-018-04448-6 (PMC5970234; doi:10.1038/s41467-018-04448-6)
Supplement: Supplementary file 3 — Description of Additional Supplementary Files [file 41467_2018_4448_MOESM3_ESM.pdf]

Description of Additional Supplementary Files

File Name: Supplementary Data 1
Description: Clinical information for discovery and validation cohorts

File Name: Supplementary Data 2
Description: Summary of molecular markers

File Name: Supplementary Data 3
Description: Whole exome sequencing information

File Name: Supplementary Data 4
Description: Summary of the somatic coding mutations (by WES) in the discovery cohort of *TERTp*^{WT}-*IDH*^{WT} gliomas (n=25)

File Name: Supplementary Data 5
Description: Summary of the copy number alterations (by WES) in the discovery cohort of *TERTp*^{WT}-*IDH*^{WT} gliomas (n=25)

File Name: Supplementary Data 6
Description: Significantly mutated genes from discovery cohort WES, IntOgen analysis

File Name: Supplementary Data 7
Description: Whole genome sequencing information (n=9)

File Name: Supplementary Data 8
Description: Summary of the somatic coding mutations (by WGS) in the discovery cohort of T*ERTp*^{WT}-*IDH*^{WT} gliomas (n=9)

File Name: Supplementary Data 9
Description: Summary of the somatic copy number variants (CNV by WGS) in the discovery cohort of *TERTp*^{WT}-*IDH*^{WT} gliomas (n=9)

File Name: Supplementary Data 10
Description: Summary of the somatic structural variants (SV by WGS) in the discovery cohort of *TERTp*^{WT}-*IDH*^{WT} gliomas (n=9)

File Name: Supplementary Data 11
Description: CRISPR/Cas9 sgRNAs, cloning oligos, and Surveyor primers

File Name: Supplementary Data 12
Description: SMARCAL1 knockout isogenic cell lines generated by CRISPR/Cas9 gene editing

File Name: Supplementary Data 13
Description: PCR programs used in study

File Name: Supplementary Data 14
Description: Primers used in this study
